# The CAREPAL-8: a short screening tool for multidimensional family caregiver burden in palliative care

**DOI:** 10.1186/s12904-024-01480-w

**Published:** 2024-08-02

**Authors:** Anneke Ullrich, Corinna Bergelt, Gabriella Marx, Anne Daubmann, Gesine Benze, Julia Heine, Lisa-Marie Dickel, Feline Wowretzko, Youyou Zhang, Carsten Bokemeyer, Friedemann Nauck, Karin Oechsle

**Affiliations:** 1 Palliative Care Unit, Department of Oncology, Hematology and BMT, University Medical Center Hamburg-Eppendorf, Martinistr. 52, 20246 Hamburg, Germany; 2 Department of Medical Psychology, University Medical Center Hamburg-Eppendorf, Hamburg, Germany; 3 Department of Medical Psychology, University Medicine Greifswald, Greifswald, Germany; 4 Department of Palliative Medicine, University Medical Center Goettingen, Goettingen, Germany; 5 Department of General Practice / Primary Care, University Medical Center Hamburg-Eppendorf, Hamburg, Germany; 6 Department of Medical Biometry and Epidemiology, University Medical Center Hamburg-Eppendorf, Hamburg, Germany

**Keywords:** Screening, Family caregiver, Palliative care, Caregiver burden, Latent class analysis

## Abstract

**Background:**

Family caregivers of terminally ill and dying people do not only experience varying levels but also different dimensions of caregiver-related strain and burden. The aim of the study was to develop a short multidimensional screening tool for the detection of burden in family caregivers in palliative care.

**Methods:**

Family caregivers of cancer patients newly admitted to specialist inpatient palliative care (*N* = 232) completed questionnaires on psychological burden, quality of life, social support and need fulfillment. A latent class mixture model was used to identify discrete classes of family caregivers related to their multidimensional caregiver burden. Multinomial logistic regression analyses were performed to identify the most predictive items from a set of established questionnaires.

**Results:**

Four latent classes of family caregivers were identified: *Currently stable caregivers* (37%), *Caregivers with unmet needs* (20%), *Psychologically burdened caregivers* (30%), and *High-risk caregivers* (13%). Each of these classes describes a different risk profile of multidimensional family caregiver burden, although family caregivers exhibit high levels of distress across all classes. From a set of 48 items, we identified eight items that predicted the class membership best. These items represent the items of the novel multidimensional screening tool: The *8*-item Screening Tool for Family *Care*giver Burden in *Pal*liative Care (CAREPAL-8). Except for social support, the items maintained fidelity to the conceptualization of multidimensional caregiver burden used in this study. A preliminary classification system was developed, which has yet to be validated.

**Conclusions:**

This study represents the first step in the establishment of a practical, self-administered screening tool that might help healthcare providers to tailor caregiver care according to their burden in daily practice. Brevity of the 8-item tool might facilitate its use in routine clinical care.

**Supplementary Information:**

The online version contains supplementary material available at 10.1186/s12904-024-01480-w.

## Background

The terminal illness of a beloved person is a major life crisis causing severe stress to family caregivers. Palliative care research demonstrates that family caregivers are confronted with multiple stressors during the disease trajectory [1, 2]. These include reduced mental and physical quality of life [3–5], physical and emotional burden [6], insomnia and sleep problems [5], anxiety, depression and other psychological morbidity [3, 7–9], and severe distress [3]. Further, the social and economic impact of caregiving can be profound: family caregivers report reduced social support [10], changes in family functioning [11], family conflicts [12, 13], and financial and job pressure [14]. Ethical dilemmas may occur in relation to decision-making responsibilities and outcomes [15, 16]. Caregiving at the end of life is intertwined with deep emotions, like feelings of failure, guilt and regret [17–19], and many caregivers are unable to reconcile.

These burdens can result in significant mental health problems among family caregivers in palliative care: The prevalence of significant anxiety is approximately 40 to 42%, while the prevalence of significant depression spans a wide range of 16 to 67% [2]. As a consequence, family caregivers with heightened psychological burden show worse levels of adjustment, social and occupational functioning [20] and are more likely to suffer from complicated grief after the patient’s death [21, 22]. In addition, if psychological burden stays undetected it can contribute to the development of chronic mental disorders [7]. However, distinguishing suspected depressive and anxiety disorders from emotions that are expected in family caregivers facing a life-threatening illness or the approaching death of a loved one can be a challenge for palliative and hospice care providers.

Further, it is well documented that family caregivers experience a wide range of care needs; however, these largely remain unmet [23–25]. A review on unmet needs in family caregivers of advanced cancer patients demonstrated that specifically information needs are often not adequately addressed [23]. Unmet needs can contribute to higher levels of family caregiver burden including poorer family caregiver health and psychological distress [26, 27]. However, meeting the support needs of family caregivers of terminally ill and dying patients remains challenging for healthcare providers [28]. Barriers include family-related challenges (e.g. poor family functioning, incongruence of family caregiver and patient needs), health system barriers (e.g. frameworks for conceptualizing family caregiving), and communication-related barriers (e.g. timing and amount of information, family caregivers not wanting to bother healthcare professionals, language barriers) [28, 29].

Family caregivers are an integral part of the ‘unit of care’ in the palliative and hospice context [30]. They are recognized not only as caregivers, but also as people who are affected by the patient’s illness and who have their own needs for support [2]. Given the serious consequences of increased psychological burden, deteriorated quality of life and unmet needs, it is evident that palliative and hospice services should use tools for the routine screening of family caregivers to discover caregiver burden at an early stage. Screening could be the first step in preventing severe consequences by identifying family caregivers in need for intensified support. Some instruments for the assessment of family caregivers have been developed for the palliative care context; in addition, generic instruments or instruments developed in other medical fields have been validated for this population of family caregivers [31–33]. These include screening tools to detect psychosocial distress (e.g. the Distress Thermometer [34]) and caregiver burden (e.g. a short screening version of the Zarit Burden Interview [35]) in family caregivers.

Available screening tools are based on constructs that include physical, psychological, social, and spiritual aspects of burden, thus covering many relevant dimensions of caregiver burden. However, they either focus on one specific dimension of burden. In order to gain knowledge about combinations or patterns of burden, several tools need to be administered to family caregivers simultaneously. The length of screening may be burdensome for this vulnerable group, and may also be impractical for clinical use. Other tools include multiple dimensions of family caregiver burden, but do not provide information on specific combinations and patterns of burden to target interventions according to need.

Taken together, multiple aspects contribute to family caregiver burden in palliative care, which is therefore characterized as a multidimensional rather than a unidimensional phenomenon [20, 36, 37]. However, to date, there is no uniform conceptualization or definition of caregiver burden in the literature, predominantly because it is so multi-faceted [38]. Further, available screening instruments are not designed to elicit distinctive combinations or patterns of burden, which could help clinicians to better support family caregivers according to their specific presentations of burden.

Thus, we developed a short multidimensional screening tool based on existing instruments that targets essential dimensions of family caregivers’ burden and is suitable for implementation in daily clinical care. Therefore, we first clarified how family caregivers of advanced cancer patients admitted to a specialist palliative care ward might be sorted into clinically relevant groups with respect to their risk profiles of multidimensional caregiver burden. Second, we identified a set of predictors for group membership, which represent the items of the novel screening tool. Third, we developed a preliminary classification system for interpreting the screening results.

## Methods

### Approach to the development of the screening tool

**Fig. 1:**
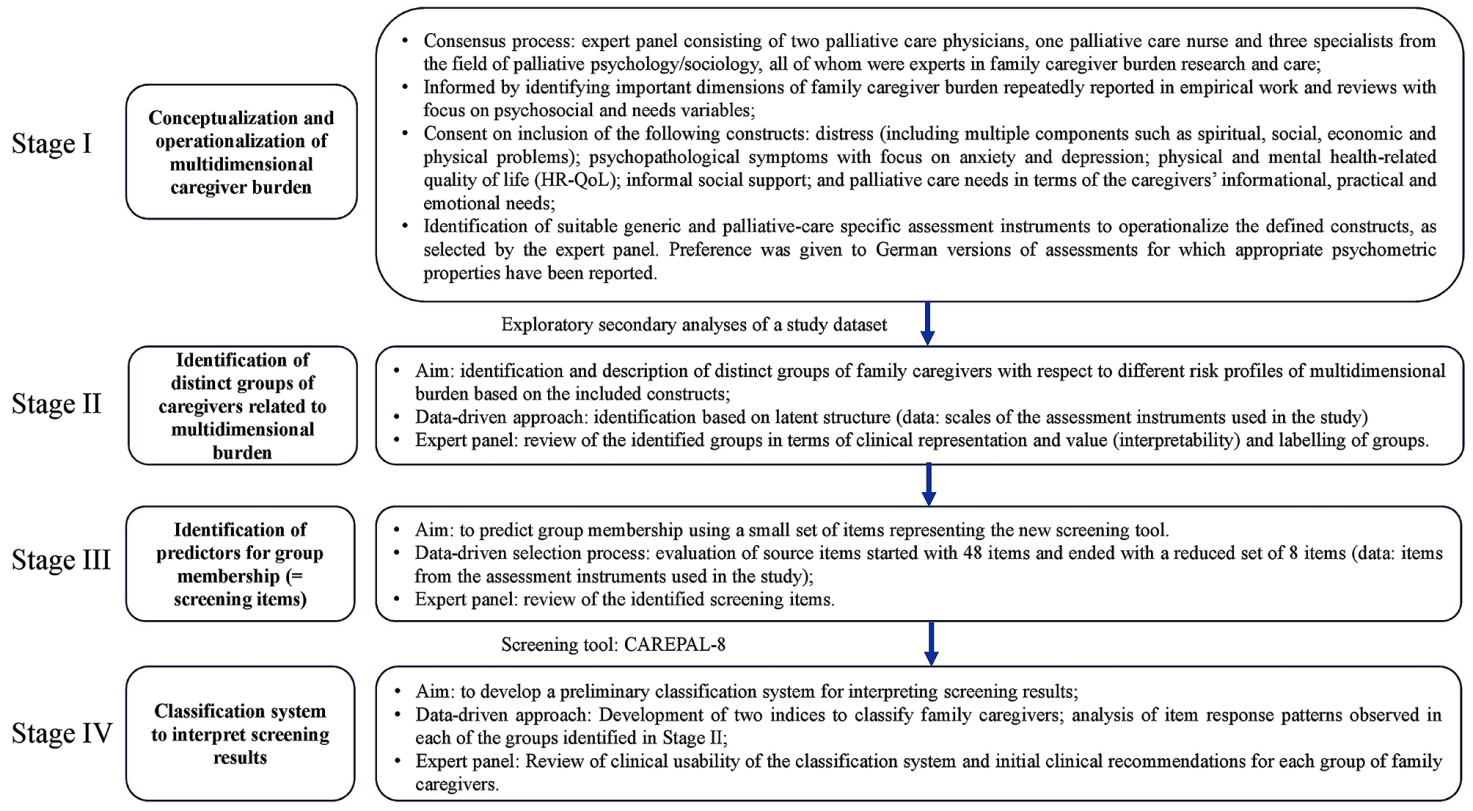
Major stages of developing the screening tool

The development of the screening tool relies on data collected in an observational study on family caregiver burden in palliative care (see ‘Data Source: The family caregiver survey in specialist inpatient palliative care’). The screening tool should capture psychosocial and needs variables relevant to family caregiver burden, be useful in routine palliative care, be brief and easy to score, and be non-commercial. Our approach to developing the screening tool is shown in Fig. 1. We briefly summarize the different stages of the development process: We began with the conceptualization and operationalization of multidimensional family caregiver burden in the design of the study, involving a panel of experts (Stage I). Once the study was conducted, secondary analyses of the dataset were performed to identify distinct groups of family caregivers in terms of their risk profiles of ‘multidimensional family caregiver burden’. The clinical representation and value of these groups were validated by a panel of experts (Stage II). In order to identify predictors for the family caregivers’ risk profiles, source items (from the assessment instruments used in the study) were evaluated. We started with 48 source items, aiming at ending with a small number of items suitable for screening (Stage III). On the basis of the item response patterns observed in each of the family caregiver groups identified in Stage II (risk profiles of ‘multidimensional family caregiver burden’), a preliminary classification system for interpreting the screening results was developed, together with initial clinical recommendations (Stage IV).

### Data source: the family caregiver survey in specialist inpatient palliative care

To develop the screening tool, an existing set of questionnaire data was analyzed from a prospective observational multicenter study designed to explore family caregiver burden in the context of specialist inpatient palliative care. Participants of this study consisted of family caregivers of advanced cancer patients consecutively recruited in 2016—2017 at the palliative care wards of two University Medical Centers in Northern Germany. Inclusion criteria were: being the primary caregiver (as indicated by the patient) and being at least 18 years of age. A priori exclusion criteria were: imminent death of the patient, legal guardianship without personal relationship to the patient, and inadequate language skills or insufficient cognitive function to complete questionnaires (as assessed by study staff). Participants completed a set of self-report questionnaires on distress, anxiety, depression, mental and physical health-related quality of life (HR-QoL), informal social support and palliative care need fulfillment within 72 h after the patient’s admission to the study ward. A detailed description of the study including methodology and data on participant inclusion appears elsewhere [3, 25]. Both the Ethics Committee of the General Medical Council Hamburg (PV5122) and the institutional Ethics Board of the University Medical Center Goettingen (1/4/16) approved the study protocol. Written informed consent was obtained from all family caregivers.

### Measurement of multidimensional caregiver burden

A set of questionnaires for assessing multidimensional caregiver burden in specialist inpatient palliative care was administered in the underlying study, including a pilot phase to test its feasibility [39]. Different assessment instruments were used to operationalize the multidimensional burden as defined in our study. These comprised standardized generic and palliative care-specific assessments with regard to psychological burden, mental and physical HR-QoL, social support and unmet palliative care needs of family caregivers. All assessment instruments are frequently used in family caregiver research, although most generic instruments have not been formally validated in family caregivers in palliative care yet [33, 40].

#### Psychological burden

To represent psychological burden, the constructs distress, anxiety and depression were selected. *(1) Distress*: The construct of distress is characterized as a multifactorial unpleasant emotional experience of psychological, social, physical and/or spiritual nature [41]. The *Distress Thermometer* (DT), originally developed for cancer patients, measures such distress within the last week on a visual analogue scale (VAS) rated from 0 “no distress” to 10 “extreme distress” [42]. For detection of clinically relevant distress with need of professional support, a cut-off value of ≥ 5 has been validated, both for patients [42] and family caregivers [34]. *(2) Anxiety and depression*: The construct of anxiety is based on core symptoms of generalized anxiety disorders as classified in the Diagnostic and Statistical Manual of Mental Disorders, 5th edition (DSM-V), including feeling nervous, anxious or on edge, not being able to stop or control worrying, and feeling afraid as if something might happen. The construct of depression is based on DSM-V criteria for depressive disorders, including depressed mood and anhedonia. The *Patient Health Questionnaire – 9-item Depression Module* (PHQ-9) [43] and *General Anxiety Disorder 7-item Scale* (GAD-7) [44], assess symptoms of depression and generalized anxiety disorder within the past two weeks. Although the development of both measures was based upon DSM-IV criteria, they are also comparable with the more recent version of DSM-V. Differences in diagnostic criteria are minimal and the use of GAD-7 and PHQ-9 is still recommended [45, 46]. In both measures, items are scored on a four-point Likert scale rated from “not at all” to “nearly every day” with a total score ranging from 0 to 27 for PHQ-9 and 0 to 21 for GAD-7. To determine prevalence of suspected depressive or anxiety disorder, a cut-off score of ≥ 10 is used.

#### Health-related quality of life

The construct of HR-QoL refers to an individual’s health and well-being in terms of general health, physical and emotional functioning and role limitations. Hence, it includes physical and mental aspects [47]. The *SF-8*, a short form of the Health Survey Form-36, was used to measure generic HR-QoL [48, 49]. Eight aspects, representing physical and mental well-being, are rated on single item scales with scores being linearly transformed to 0—100, with higher values representing better outcomes.

#### Social support

The construct of social support refers to the availability of informal support from the family caregiver’s social network when requested [50, 51]. Availability of such support was measured by the generic *OSLO-3-Items-Social-Support-Scale* (OSLO-3) ranging from 3 to 14 with categorization into poor (3–8), moderate (9–11) and strong (12–14) support [50].

#### Unmet palliative care needs

The construct of fulfillment of needs refers to palliative care needs that family caregivers experience when accompanying a terminally ill or dying person, which, in the family caregivers’ perception, may be met or unmet [52]. The 20-item *Family Inventory of Needs* (FIN), a palliative care-specific measure, was used to assess the number and nature of unmet needs [52, 53]. Needs are rated “not met”, “partly met”, and “met”. Breadth scores of unmet needs, defined as not or only partly met needs, were calculated for four domains of the FIN [54]: Basic information (score: 0—4), Information on treatment and care (score: 0—7), Support (score: 0—7) and Patient comfort (score: 0—2). Higher values represent more unmet needs.

### Data analysis

Due to the exploratory nature of the data analysis [55], sample size calculations were not performed. The same study sample was used for all analyses, and constructs served as both dependent and independent variables but at different levels of data aggregation (scale level vs. item level). All significance tests were two-sided using a significance level of α < 0.05.

Latent class mixture modeling was used as a first step in the development of the new screening tool. The aim was the data-driven identification of distinct groups of caregivers in terms of their risk profile of multidimensional family caregiver burden. Latent class indicators were the 16 scales of the assessment instruments (categorized or continuous) used to operationalize multidimensional burden: DT: VAS dichotomized as per cut-off (categorical); PHQ-9: total score dichotomized as per cut-off (categorical); GAD-7: total score dichotomized as per cut-off (categorical); SF-8: 8 single-item scales (continuous); OSLO-3: total score (continuous); FIN: 4 sub-scale scores (continuous). A complete case analysis was applied. Goodness-of-fit statistics were used to select the optimal model. We compared successive models by the Bayesian Information Criterion (BIC) and the Akaike Information Criterion (AIC) [56]. As recommended, statistical criteria were evaluated in conjunction with interpretability [57]. In the selected model, each class was assigned a label based on the constructs that characterized the classes relative to each other. Latent class mixture modeling was conducted using Mplus version 6.11 software [58].

In order to identify a small number of items suitable for screening, multinomial logistic regression analyses were performed with group membership as the dependent variable. Independent variables were the 48 source items from the assessment instruments used: DT: 1 item; PHQ-9: 9 items; GAD-7: 7 items; SF-8: 8 items; OSLO-3: 3 items; FIN: 20 items. Exploratory data analysis (e.g. cross-tabulation) was conducted to check data quality of the independent variables. Possible multicollinearity of independent variables was tested by correlational analysis (Spearman’s r), variance inflation factors (VIFs) and tolerance indices (TIs). Odds ratios (OR) with 95% confidence intervals (CI) were used as measure of association between the independent variables and the outcome. Stepwise backwards-selection and listwise deletion of missing values were applied in all regression analyses.

Because of the large set of independent variables and the given sample size [59], we used a hierarchical multi-step approach for variable selection: To pre-select predictors per dimension, four multiple regression analyses were conducted to examine the relationship between items representing psychological burden, mental and physical HR-QoL, social support, and unmet palliative care needs, and group membership. Thus, a separate regression analysis was conducted for each of the four family caregiver burden dimensions (= unidimensional approach). Subsequently, all predictors that showed a significant relationship were entered into a superordinate multiple regression analysis (= multidimensional approach) to identify a set of items that showed the highest predictive power for group membership. Analyses were conducted using the statistical package SPSS version 24.0 (IBM, USA).

The development of the preliminary classification system for interpreting the screening results based on a data-driven approach: Indices were constructed based on the risk profiles of multidimensional caregiver burden as identified by the latent class mixture models. We analysed item response patterns of the different groups of family caregivers. Therefore, means and percentages of the selected screening items were evaluated and dichotomous scores of 0 (= no burden) or 1 (= burden) were assigned to each item. Using the developed system, family caregivers were classified based on empirical data to test whether they were classified in the same group as the latent class estimate. However, it has to be noted that class assignment in latent class mixture models is based on probabilities; therefore, the classification system remains preliminary until validation.

## Results

### Study recruitment and sample characteristics

Of 693 patients admitted to the palliative care wards, 438 primary family caregivers were eligible and were approached for study participation. 81% of those who agreed to participate (232 of 287 family caregivers) responded to the questionnaire. Study participants were mid-age adults (mean age 55.5 years), 66% were female and 64% were a spouse/partner of the cancer patient. More than one third (39%) had attained a school graduation qualifying for university entrance. 43% of patients had been diagnosed in the preceding year. Table 1 gives an overview of key characteristics of family caregivers and patients. Detailed information on sample characteristics were published elsewhere [3].

**Table 1:** Sample characteristics (N = 232)

*Family caregiver characteristics*		
Age, M (SD); Range		55.5 (14.8); 20–88
Gender, n (%)	Male	78 (33.6)
	Female	154 (66.4)
Relationship to the patient. The patient is…, n (%)	Spouse/partner	148 (63.8)
	Mother/father	61 (26.3)
	Others ^a^	23 (9.9)
Having Children, n (%)	Yes	164 (70.7)
	No	24 (27.6)
	Missing	4 (1.7)
Educational level, n (%)	Low (≤ 9 years)	65 (28.0)
	Medium (10 years)	72 (31.0)
	High (12–13 years) ^b^	91 (39.2)
	Missing	4 (1.7)
Working situation, n (%)	Working	123 (53.0)
	Not working	99 (42.7)
	Missing	10 (4.3)
*Patient- and care-related characteristics*		
Gender, n (%)	Male	118 (50.9)
	Female	105 (45.3)
	Missing	9 (3.9)
Age, n (%)	≤ 60 years	75 (32.3)
	> 60 years	155 (66.8)
	Missing	2 (0.9)
Time from cancer diagnosis to admission to the palliative care ward, n (%)	≤ 12 months	99 (42.7)
	> 12 months	125 (53.9)
	Missing	8 (3.4)
Place of care before admission to the palliative care ward, n (%)	Home care without any nursing service	82 (35.3)
	Home care with nursing service not specialized in palliative care	21 (9.1)
	Home care with nursing service specialized in palliative care	33 (14.2)
	Hospital wards	82 (35.3)
	Others ^c^	11 (4.8)
	Missing	3 (1.3)
Involvement of family caregiver in physical patient care before admission to the palliative care ward, n (%)	Yes	107 (46.1)
	No	118 (50.9)
	Missing	7 (3.0)
Advance directives: patient decree, n (%)	Yes	140 (60.3)
	No	92 (39.7)
Advance directives: power of attorney, n (%)	Yes ^d^	159 (68.5)
	No	73 (31.5)

*Abbreviations* M, Mean; SD, Standard deviation

^a^ Adult children, siblings, close friends or other relatives; ^b^ Qualifying for university entrance; ^c^ Nursing home in 9 patients or other care facilities; ^d^ The patient had appointed the family caregiver to act as substitute decision-maker in terms of personal matters (including health) if needed

### Latent classes of multidimensional caregiver burden

**Fig. 2:**
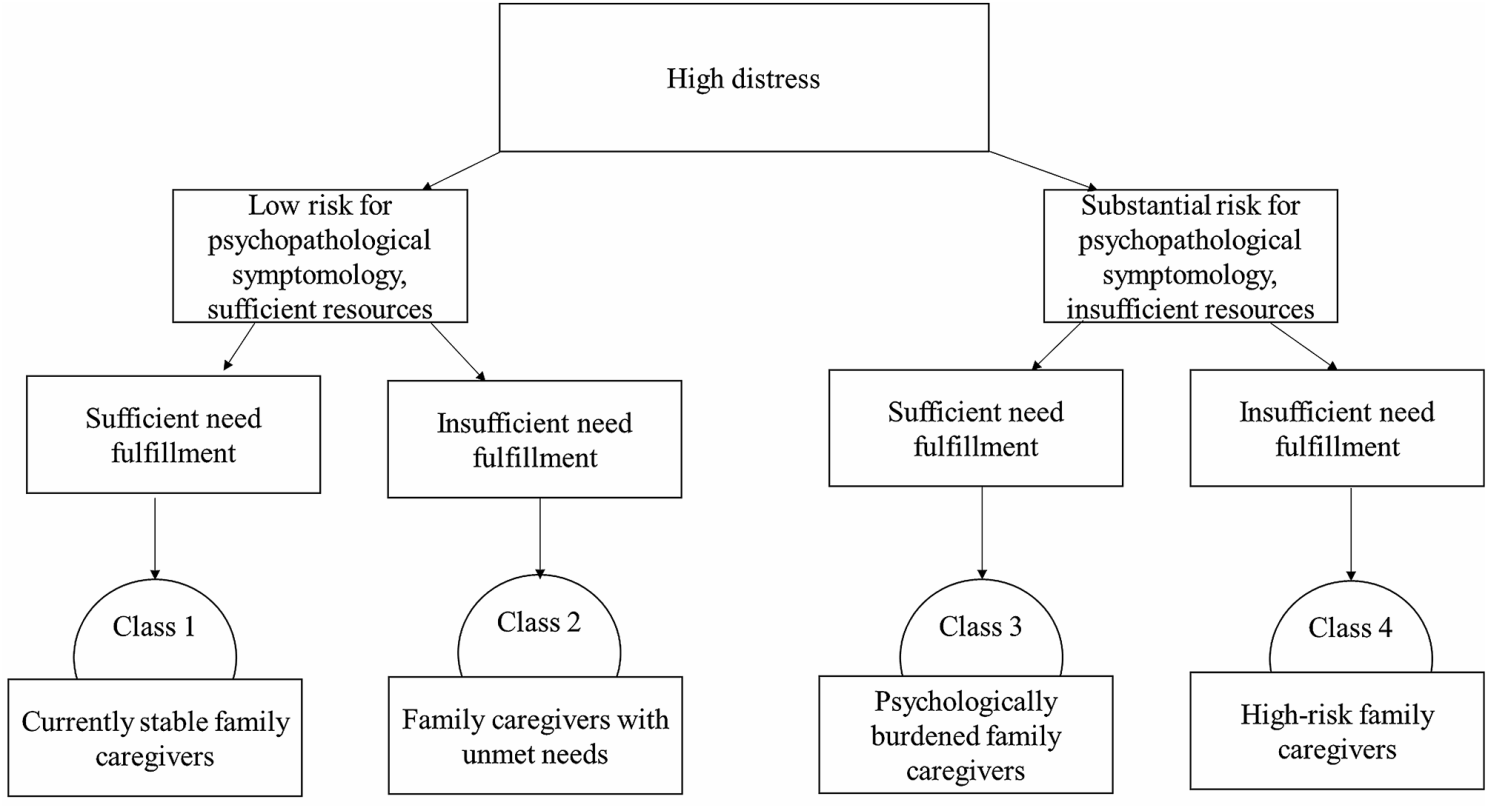
Model of family caregiver classes related to multidimensional family caregiver burden

The latent class mixture models were fit to respondents with complete data (*N* = 225; 97% of the sample). Table 2 presents the goodness-of-fit indices for all five tested models. Based on the statistical analysis, the expert panel of palliative care and psychosocial specialists interpreted class solutions theoretically, proved whether small classes (< 10%) made conceptual sense, and elaborated the implications of class membership for clinical practice. In combination with clinical interpretation, model fit criteria indicated that a 4-class model was superior to 1-, 2-, and 3-class-models. When testing 4- versus 5-class-models, the gain of the added class was small and substantive clinical interpretation supported the 4-class-model, because this solution yielded clearer application to practice. According to the reported fit indices and further parameters (e.g. posterior probabilities, not shown), the selected model was stable and valid. With regard to multidimensional caregiver burden, the following four classes of family caregivers emerged (Fig. 2). Descriptive results for each class are represented in Table 3. These descriptions are based on the complete sample of family caregivers (*N* = 232):

- *Currently stable family caregivers (class 1)*. Family caregivers in this class (*n* = 86 of 232, 37%) exhibit low prevalence of psychopathological symptoms, yet are highly distressed. They possess relatively sufficient resources with respect to mental and physical HR-QoL and social support. Need fulfilment is sufficient across all domains.
- *Family caregivers with unmet needs (class 2)*. Specifically, family caregivers in this class (*n* = 46 of 232, 20%) report substantial levels of unmet needs across all domains. The majority shows low prevalence of psychopathological symptoms; however, distress is high. Related to mental and physical HR-QoL and social support, relatively sufficient resources are available. Nevertheless, low social support gains relevance in this class.
- *Psychologically burdened family caregivers (class 3)*. Caregivers in this class (*n* = 70 of 232, 30%) reveal, beyond distress, relevant psychopathological symptoms. Resources are insufficient, as mental and physical HR-QoL is severely impaired and social support is diminished. However, despite their psychological burden and limited resources, family caregivers of this class report sufficient need fulfillment.
- *High-risk family caregivers (class 4)*. Family caregivers in this class (*n* = 30 of 232, 13%) exhibit the highest prevalence of psychopathological symptoms and distress, combined with insufficient resources due to severely impaired mental and physical HR-QoL and poor social support as well as unmet needs across all domains.

Taken together, all family caregivers show high distress. While high psychological burden corresponds with insufficient availability of resources (class 3 and 4), less psychological burden corresponds with sufficiency of resources (class 1 and 2). However, deficiencies in need fulfillment are reported in both conditions (class 2 and 4). Figure 2 depicts the most distinguishing aspects of multi­dimensional caregiver burden related to the four identi­fied classes.

**Table 2:** Model fit indices for latent classes of multidimensional family caregiver burden

Class	Log-Likelihood	Free Parameter	AIC	BIC	Entropy
1	-8519	29	17093	17193	-
2	-8190	47	16474	16636	0.906
3	-8024	65	16177	16401	0.918
4	-7884	83	15935	16221	0.920
5	-7840	101	15883	16231	0.903

*Abbreviations* AIC, Akaike information criterion; BIC, Bayesian information criterion

### Multivariable predictor selection

The predictor selection process started with 48 source items from the assessment instruments used in the study and ended with a reduced set of eight items, representing the screening items. Four unidimensional multiple regression analyses were conducted on (1) 17 psychological burden variables (distress, anxiety and depression), (2) 8 HR-QoL variables, (3) 3 informal social support variables, and (4) 20 palliative care need variables to examine the relationships with the four family caregiver groups. The results of these unidimensional regression analyses are not shown but are available from the first author upon reasonable request. In the first step of the superordinate multidimensional regression model, 14 variables that had shown a significant relationship in the unidimensional regression analyses were included. Stepwise backwards-selection showed the following eight items to be the optimal predictor set for class membership (Table 4):

- Not able to stop worrying (item #2, GAD-7).
- Feeling tired or lacking energy (item #4, PHQ-9).
- Bodily pain (item #4, SF-8).
- Absence of daily activities because of emotional problems (item #8, SF-8).
- Need to know what treatment the patient is receiving (item #6, FIN).
- Need to know what symptoms the treatment or disease can cause (item #11, FIN).
- Need to have information about what to do for the patient at home (item #16, FIN).
- Need to feel accepted by the health professionals (item #17, FIN).

These eight items present the novel *8*-item Screening Tool for Family *Care*giver Burden in *Pal*liative Care (CAREPAL-8). Table 5 shows the description of selected screening items stratified by the four classes of family caregivers. The set of items, including the question and response options, can be found in the supplemental material (Figure A1). It should be noted that the CAREPAL-8 is based on source items from established instruments (GAD-7, PHQ-9, SF-8, and FIN). No permissions or rights had to be obtained for the GAD-7 and PHQ-9 as they are available without copyright restrictions for non-commercial use. The SF-8 is publicly available for use in research by non-commercial users. Permission to use the FIN for our purposes was obtained from the authors.

**Table 3:** Description of groups stratified by latent class analysis (N = 232)

Outcomes	Class 1: Currently stable family caregivers (n = 86)	Class 2: Family caregivers with unmet needs (n = 46)	Class 3: Psychologically burdened family caregivers (n = 70)	Class 4: High-risk family caregivers (n = 30)
**Psychological impairments**				
Distress (DT), n (%)				
No clinically relevant distress (< 5)	7 (8.1)	1 (2.2)	2 (2.9)	1 (3.3)
Clinically relevant distress (≥ 5)	79 (91.9)	45 (97.8)	68 (97.1)	29 (96.7)
Anxiety (GAD-7), n (%)				
None/mild symptoms (< 10)	67 (77.9)	28 (60.9)	20 (28.6)	3 (10.0)
Moderate/severe symptoms (≥ 10)	17 (19.8)	15 (32.6)	49 (70.0)	25 (83.3)
Missing	2 (2.3)	3 (6.5)	1 (1.4)	2 (6.7)
Depression (PHQ-9), n (%)				
None/mild symptoms (< 10)	77 (89.5)	35 (76.1)	20 (28.6)	7 (23.3)
Moderate/severe symptoms (≥ 10)	7 (8.2)	10 (21.7)	48 (68.5)	22 (73.4)
Missing	2 (2.3)	1 (2.2)	2 (2.9)	1 (3.3)
**Quality of Life (SF-8; 0-100)**, M (SD) ^a^				
General Health (GH)	46.8 (5.2)	45.9 (5.5)	37.3 (6.0)	36.7 (6.3)
Physical Functioning (PF)	51.3 (4.1)	50.7 (4.6)	39.6 (7.9)	37.3 (8.3)
Role Physical (RP)	49.9 (5.9)	50.6 (4.9)	37.1 (8.1)	30.2 (6.1)
Bodily Pain (BP)	56.7 (7.2)	57.2 (7.1)	46.7 (11.8)	44.4 (10.2)
Vitality (VT)	49.5 (7.0)	48.5 (7.5)	41.3 (7.2)	38.3 (7.4)
Social Functioning (SF)	48.7 (8.0)	47.0 (9.7)	40.6 (10.5)	38.0 (10.3)
Mental Health (MH)	46.4 (7.9)	39.4 (9.8)	34.6 (9.6)	35.2 (9.3)
Role Emotional (RE)	44.4 (8.2)	43.7 (8.0)	34.6 (8.6)	30.4 (6.8)
**Availability of informal social support (OSLO-3)**, n (%)				
Poor (3—8)	5 (5.8)	11 (23.9)	15 (21.4)	12 (40.0)
Moderate (9—11)	38 (44.2)	12 (26.1)	30 (42.9)	10 (33.3)
Strong (12—14)	43 (50.0)	22 (47.8)	25 (35.7)	8 (26.7)
Missing	0 (0.0)	1 (2.2)	0 (0.0)	0 (0.0)
**Number of unmet needs (FIN)**, M (SD) ^a^				
Basic information (0—4)	0.8 (0.9)	2.4 (1.1)	0.7 (1.0)	2.1 (1.2)
Information on treatment and care (0—7)	2.0 (2.0)	5.1 (1.8)	1.6 (1.8)	4.8 (2.2)
Support (0—7)	2.4 (1.7)	4.8 (1.7)	2.9 (1.5)	4.9 (1.9)
Patient comfort (0—2)	0.0 (0.0)	1.0 (0.0)	0.0 (0.0)	1.1 (0.3)

*Abbreviations* DT, Distress Thermometer; GAD-7, Generalized Anxiety Disorder Scale; PHQ-9, Patient Health Questionnaire – depression module; SF-8, short form of the Health Survey Form-36; OSLO-3, OSLO-3-Items-Social-Support-Scale; FIN, Family Inventory of Needs

^a^ n correlates to n presented for classes

**Table 4:** Multidimensional factors related to family caregiver class. Multinomial logistic regressions for *Family caregivers with unmet needs* (class 2), *Psychologically burdened family caregivers* (class 3) and *High-risk family caregivers* (class 4) with respect to *Currently stable family caregivers* (class 1)

Independent Variable	OR (95% CI) Class 2	OR (95% CI) Class 3	OR (95% CI) Class 4
**Anxiety**			
Not able to stop worrying	1.621 (0.724—3.630)	4.514 (1.911—10.661)**	4.179 (1.350—12.931)*
**Depression**			
Feeling tired or lacking energy	0.401 (0.156—1.031)	2.923 (1.278—6.684)*	1.184 (0.388—3.616)
**Quality of Life**			
Bodily Pain	0.843 (0.421—1.687)	2.950 (1.661—5.238)***	4.524 (2.107—9.714)***
Absence from daily activities because of emotional problems	1.361 (0.727—2.550)	3.005 (1.585—5.695)**	9.123 (2.892—28.782)***
**Unmet Needs**			
Know what treatment the patient is receiving	0.238 (0.066—0.859)**	2.114 (0.349—12.799)	0.051 (0.005—0.468)**
Know what symptoms the treatment or disease can cause	0.326 (0.056—1.898)	10.801 (0.936—24.596)	0.732 (0.052—10.387)
Have information about what to do for the patient at home	0.080 (0.015—0.428)**	0.489 (0.109—2.203)	0.397 (0.038—4.131)
Feel accepted by the health professionals	0.087 (0.010—0.770)*	0.033 (0.003—0.414)*	0.057 (0.003—1.026)

Reference group: *Currently stable family caregivers* (class 1); Nagelkerke’s Pseudo R^2^ = 0.806

*p < .05, **p < .01, ***p < .001

**Table 5:** Description of screening items stratified by family caregiver classes (N = 232)

	Class 1: Currently stable family caregivers (n = 86)	Class 2: Family caregivers with unmet needs (n = 46)	Class 3: Psychologically burdened family caregivers (n = 70)	Class 4: High-risk family caregivers (n = 30)
**Anxiety** ^a, b^				
Not able to stop worrying (Scale: 0—3), M (SD)	0.65 (0.736)	0.98 (0.897)	1.55 (1.019)	1.72 (0.996)
**Depression** ^a, b^				
Feeling tired or lacking energy (Scale: 0—3), M (SD)	1.04 (0.070)	1.13 (0.778)	2.19 (0.902)	2.24 (8.72)
**Quality of Life** ^b, c^				
Bodily Pain (Scale: 1—6), M (SD)	1.58 (1.034)	1.50 (1.006)	3.00 (1.654)	3.33 (1.422)
Absence from daily activities because of emotional problems (Scale: 1—5), M (SD)	2.12 (1.080)	2.20 (1.057)	3.35 (1.082)	3.89 (0.832)
**Unmet Needs** ^d^				
Know what treatment the patient is receiving, n (%) unmet	15 (19.2)	30 (69.8)	6 (9.7)	18 (75.0)
Know what symptoms the treatment or disease can cause, n (%) unmet	4 (5.5)	18 (41.9)	4 (5.7)	9 (36.0)
Have information about what to do for the patient at home, n (%) unmet	25 (38.7)	31 (88.6)	20 (40.0)	14 (73.7)
Feel accepted by the health professionals, n (%) unmet	2 (2.7)	10 (26.3)	6 (9.7)	9 (39.1)

^a^ Range of the response options (Likert-scales): 0=”not at all” to 3=”nearly every day”; ^b^ n correlates to n presented for classes; ^c^ Range of the response options (Likert-scales); bodily pain: 1=”none” to 6=”very severe”, absence from daily activities because of emotional problems: 1=”not at all” to 5 “could not do daily activities”; ^d^ answers are dichotomized in “not met” (partly/not) and “met”. Presented data reflects the number and proportion of patients whose needs were unmet

### Preliminary classification system for interpreting the screening results

The CAREPAL-8, a self-administered screening tool, can be used to stratify family caregivers with regard to multidimensional caregiver burden. For this purpose, a preliminary classification system was developed that reflects the four identified classes of family caregivers. To stratify the family caregivers, two indices were built – one index to distinguish between family caregivers with low psychopathological symptoms and sufficient resources versus those with substantial psychopathological symptoms and insufficient resources (index 1: PSYQOL), and the other to differentiate between sufficient versus insufficient fulfilment of palliative care needs (index 2: NEEDS). Combinations of these two indices form the basis for assigning family caregivers to the four classes. The preliminary classification system is presented as supplemental material (Figure A2). Additionally, initial clinical recommendations for further family caregiver assessment and support, as developed by the expert panel, are provided as supplemental material (Figure A3).

## Discussion

### Key findings and clinical implications

It has been claimed that it is inappropriate to overburden family caregivers with unnecessarily comprehensive or multiple self-report questionnaires [31]. Further, administration of multiple, unidimensional questionnaires might be a hindrance for the implementation of routine screening of family caregivers within daily clinical practice. Therefore, we aimed to improve daily practicability by developing a screening tool with family caregivers’ absolute minimum of items. This article describes the development of the CAREPAL-8, a short screening tool to detect family caregivers’ multidimensional burden in palliative care.

We were able to show that family caregivers can be differentiated in four classes with clinically different risk profiles: *Currently stable family caregivers*, *Family caregivers with unmet needs*, *Psychologically burdened family caregivers*, and *High-risk family caregivers*. While distress was high across the four groups, we found several distinguishable differences among the identified classes. Different risk profiles of psychological burden as well as mental and physical HR-QoL and informal social support (resources) were identified. However, we saw similar patterns across the groups: *Currently stable family caregivers* and *Family caregivers with unmet needs* collectively showed the best outcomes in both psychological burden and resources. Likewise, *Psychologically burdened family caregivers* and *High-risk family caregivers* reported the most adverse outcomes in both areas. Need fulfillment appeared to further distinguish groups. Interestingly, deficiency in need fulfillment was prevalent in family caregivers both with the best and adverse outcomes regarding psychological burden and resources, namely *Family caregivers with unmet needs* and *High-risk family caregivers*. Our findings underline previous research on the relationship of psychological burden with mental and physical HR-QoL and social support among family caregivers of terminally ill and dying patients. For example, sufficient social support is well-known to have a buffering effect on family caregivers’ mental health [60, 61]. A stable psychological condition may have a positive impact on family caregivers’ mental and physical HR-QoL [62], which in turn is a protective factor for mental health problems. Our findings demonstrate that need fulfilment can be impaired regardless of the intensity of psychological burden and resources. Nevertheless, there is evidence that unmet needs negatively affect family caregivers’ psychological condition when caring for terminally ill and dying patients [23].

Overall, the clinically diverse risk profiles imply consequences for family caregivers’ care, for example the adaption of targets, intensity and providers of clinical interventions. Family caregivers whose problems focus on unmet needs might benefit from other interventions than family caregivers who struggle from severe psychological burden. For example, a short psychoeducational intervention was able to improve family caregivers’ psychological distress, but not unmet needs [63].

From a large item pool, eight items emerged to be the optimal predictor set for class membership: one item related to anxiety, one item related to lack of energy, two quality-of-life items related to bodily pain and absence from daily activities because of emotional problems, and four items related to needs. Need items reflected knowledge about the patient’s treatment, knowledge about symptoms that may occur, information about caregiving at home, and feeling accepted by healthcare providers. Thus, the selected items maintained fidelity to the concept of multidimensional family caregiver burden used in this study, except for social support. The eight selected items represent the novel, practical, self-administered tool, developed for rapid screening of family caregivers burden in daily palliative care practice. Uniquely, this tool comprises of factors from multiple dimensions of family caregiver burden to identify those at risk for psychological burden, diminished resources and unmet needs. Thus, the screening can support palliative care professionals identifying family caregivers with intensified need for support and linking them to interventions that couple with their sources of burden.

However, clinical utility as well as validity of the screening, including the proposed simple classification system for interpreting the screening results, have to be determined in future research. If the novel CAREPAL-8 proves valid, a tool would be available that would make it possible to classify family caregivers with regard to different risk profiles in daily clinical palliative care. We anticipate that such a novel screening may have significant implications for healthcare providers and policy-makers helping them to recognize, prioritize and address family caregivers’ burden. In preparation for this, initial recommendations for further assessment and support of family caregivers were developed, which can be found in the supplemental material (Figure A3). These recommendations are based on clinical expertise combined with conclusions from palliative care research on family caregivers’ psychological burden, mental and physical HR-QoL, social support and need fulfillment. Additionally, it must be kept in mind that many family caregivers in palliative care do not speak the local language, the application of non-native language versions may be difficult, and (lay) interpreting may affect the screening results. If the CAREPAL-8 proves valid, its translation to other languages, accounting for linguistic and cultural differences, should be considered.

### Limitations

Several limitations of our study should be noted. First, the cohort consists of family caregivers of cancer patients entering specialist inpatient palliative care, which limits the generalizability of the screening tool. Future work will be needed to extrapolate our findings in other samples, like family caregivers of patients with other life-limiting diseases than cancer and family caregivers in home-based hospice and palliative care settings. A second limiting factor is the number of 232 study participants, which resulted in relatively small numbers of cases in the four identified classes of family caregivers. Specifically, the class of *High-risk family caregivers* consisted of only 30 caregivers (13%). It has been suggested that 300 or more cases are desirable for latent class analysis, but smaller samples may be adequate [64]. However, the latent class mixture models performed in our study proved to be robust and valid, and classes with small memberships were uncovered. A third limitation relates to the constructs reflecting multidimensional caregiver burden, as defined in our study. As no uniform conceptualization or definition of burden exists, the inclusion and exclusion of these constructs was based on a literature review and expert consensus. As intended, selected constructs capture a range of psychosocial and needs variables; however, aspects such as spiritual issues were only superficially included, and others, such as anticipatory grief, which is a specific challenge faced by family caregivers of patients receiving palliative care, were not included at all. Nevertheless, our conceptualization comprises major constructs that have been repeatedly identified as central components of family caregiver burden [1, 2, 20, 23, 26, 37].

Notwithstanding these limitations, major strengths of the study were the multicenter design, which included study sites in an urban and a more rural area, the use of standardized, validated instruments from which screening items were derived, and our approach of identifying distinguishable classes of family caregivers.

## Conclusion

This study developed a short multidimensional screening tool that may help healthcare providers to identify family caregivers with different risk profiles of psychological burden, resources and need fulfilment. The 8-item tool is based on well-established instruments that target essential dimensions of family caregivers’ burden. Based on the screening result, family caregivers can be referred to supportive interventions with greatest benefit for alleviating the identified burden. Thus, family caregiver burden might be reduced, and healthcare providers might find guidance in caring for the family caregivers and link them with appropriate supports. Further research is required to investigate the utility and validity of the proposed screening tool as well as the classification system across family caregivers of different patient groups and settings of palliative care. An ongoing validation study is investigating the psychometric properties of the CAREPAL-8 in 16 in- and outpatient specialist palliative care facilities across Germany covering three palliative care settings (palliative care wards, hospital-based multiprofessional palliative care advisory teams, and specialist palliative home care teams). Initial recommendations should also be validated, for example using an expert consensus approach. Additionally, this study provides important information regarding multidimensional family caregiver burden. The four identified classes give insights into family caregivers’ risk profiles of psychological burden, available resources in terms of mental and physical HR-QoL and social support, and fulfilment of palliative care needs. Ultimately, this research aims to enhance the diagnostics of multidimensional family caregiver burden and the quality of support provided to family caregivers during the palliative care journey.

### Electronic supplementary material

Below is the link to the electronic supplementary material.

Supplementary Material 1

Supplementary Material 2

Supplementary Material 3

## Data Availability

The dataset supporting the conclusions of this article is available upon reasonable request from the corresponding author (AU) and with permission of the data protection officer of the University Medical Center Hamburg-Eppendorf, Hamburg, Germany.

## Abbreviations

BIC: Bayesian Information Criterion
AIC: Akaike Information Criterion
CAREPAL-8: 8-item Screening Tool for Family Caregiver Burden in Palliative Care
DSM: Diagnostic and Statistical Manual of Mental Disorders
DT: Distress Thermometer
FIN: Family Inventory of Needs
GAD-7: General Anxiety Disorder 7-item Scale
HR-QoL: Health-related quality of life
OSLO-3: OSLO-3-Items-Social-Support-Scale
PHQ-9: The Patient Health Questionnaire ? 9-item Depression Module
SF-8: 8-item short form of the Health Survey Form-36
