# Supplementary material for: The CAREPAL-8: a short screening tool for multidimensional family caregiver burden in palliative care
Source: BMC Palliat Care. 2024 Aug 2;23:195. doi: 10.1186/s12904-024-01480-w (PMC11295689; doi:10.1186/s12904-024-01480-w)
Supplement: Supplementary file 1 — Supplementary Material 1 [file 12904_2024_1480_MOESM1_ESM.pdf]

Supplemental material

The CAREPAL-8: a short screening tool for multidimensional family caregiver burden in palliative care

Figure A1: The CAREPAL-8

CAREPAL-8						
**Over the past week, how often have you been bothered by the following problems?**						
	Not at all	Several days	More than half of the days	Nearly every day		
1. Not being able to stop or control worrying	❑	❑	❑	❑		
2. Feeling tired or having little energy	❑	❑	❑	❑		
**Over the past week, how much have you been bothered by the following problems?**						
3. How much bodily pain have you had during the past week?						
	None	Very mild	Mild	Moderate	Severe	Very severe
	❑	❑	❑	❑	❑	❑
4. During the past week, how much did personal or emotional problems keep you from doing your usual work, school or other daily activities?						
	Not at all	Very little	Somewhat	Quite a lot	Could not do daily activities	
	❑	❑	❑	❑	❑	
**To which extent do you perveive that your needs are met, partly met or not met?** *If you do not have a need, please mark this need as 'met'.*						
My need to … is:	Met	Partly met	Not met			
5. know the treatment the patient is receiving	❑	❑	❑			
6. know what symptoms the treatment or disease can cause	❑	❑	❑			
7. have information about what to do for the patient at home	❑	❑	❑			
8. feel accepted by the health professionals	❑	❑	❑			

Figure A1. CAREPAL-8 screening tool for family caregivers in palliative care.

Figure A1 - Items cited from:
Item 1 (GAD-7): Spitzer RL, Kroenke K, Williams JB, Löwe B: A brief measure for assessing generalized anxiety disorder: the GAD-7. Arch Intern Med 2006, 166(10):1092-1097.
Item 2 (PHQ-9): Kroenke K, Spitzer RL, Williams JB: The PHQ-9: validity of a brief depression severity measure. J Gen Intern Med 2001, 16(9):606-613.
Items 3 and 4 (SF-8): Ware JE, Kosinski M, Dewey JE, Gandek B: How to Score and Interpret Single-Item Health Status Measures: A Manual for Users of the SF-8 Health Survey. Lincoln (RI): Quality Metric Incorporated; 2001.
Items 5 to 8 (FIN): Schur S, Neubauer M, Amering M, Ebert-Vogel A, Masel EK, Sibitz I, Watzke H, Schrank B: Validation of the Family Inventory of Needs (FIN) for family caregivers in palliative care. Palliat Support Care 2015, 13(3):485-491.

Abbreviations: GAD-7, Generalized Anxiety Disorder Scale; PHQ-9, Patient Health Questionnaire – depression module; SF-8, short form of the Health Survey Form-36; FIN, Family Inventory of Needs
