# Supplementary material for: The CAREPAL-8: a short screening tool for multidimensional family caregiver burden in palliative care
Source: BMC Palliat Care. 2024 Aug 2;23:195. doi: 10.1186/s12904-024-01480-w (PMC11295689; doi:10.1186/s12904-024-01480-w)
Supplement: Supplementary file 2 — Supplementary Material 2 [file 12904_2024_1480_MOESM2_ESM.pdf]

Supplemental material

The CAREPAL-8: a short screening tool for multidimensional family caregiver burden in palliative care

Figure A2: Preliminary classification system

**CAREPAL-8**							**Rating**
**Over the past week, how often have you been bothered by the following problems?**							**Index 1 (PSYQOL)**
		Not at all	Several days	More than half of the days	Nearly every day		
1. Not being able to stop or control worrying		❑0	❑0	❑1	❑1		☐ +
2. Feeling tired or having little energy		❑0	❑0	❑1	❑1		☐
**Over the past week, how much have you been bothered by the following problems**							
3. How much bodily pain have you had during the past week?							
None	Very mild	Mild	Moderate	Severe	Very severe		
❑0	❑0	❑0	❑1	❑1	❑1		☐ +
4. During the past week, how much did personal or emotional problems keep you from doing your usual work, school or other daily activities?							
Not at all	Very little	Somewhat	Quite a lot	Could not do daily activities			
❑0	❑0	❑0	❑1	❑1			☐ =
**Result Index 1 (PSYQOL):**							☐

**To which extent do you perceive that your needs are met, partly met or not met?** *If you do not have a need, please mark this need as 'met'.*				**Index 2 (NEEDS)**
My need to … is:	Met	Partly met	Not met	
5. know the treatment the patient is receiving	❑0	❑1	❑1	☐ +
6. know what symptoms the treatment or disease can cause	❑0	❑1	❑1	☐ +
7. have information about what to do for the patient at home	❑0	❑1	❑1	☐ +
8. feel accepted by the health professionals	❑0	❑1	❑1	☐ =
**Result Index 2 (NEEDS):**				☐

CLASSIFICATION			
**Currently stable family caregiver:** ☐	**Family caregiver with unmet needs:** ☐	**Psychologically burdened family caregiver:** ☐	**High-risk family caregiver:** ☐
Index 1 = 0<br>Index 2 ≤ 1	Index 1 = 0<br>Index 2 ≥ 2	Index 1 = 1<br>Index 2 ≤ 1	Index 1 = 1<br>Index 2 ≥ 2

Figure A2. CAREPAL-8: Preliminary classification system

Figure A2 - Items cited from:
Item 1 (GAD-7): Spitzer RL, Kroenke K, Williams JB, Löwe B: A brief measure for assessing generalized anxiety disorder: the GAD-7. Arch Intern Med 2006, 166(10):1092-1097.
Item 2 (PHQ-9): Kroenke K, Spitzer RL, Williams JB: The PHQ-9: validity of a brief depression severity measure. J Gen Intern Med 2001, 16(9):606-613.
Items 3 and 4 (SF-8): Ware JE, Kosinski M, Dewey JE, Gandek B: How to Score and Interpret Single-Item Health Status Measures: A Manual for Users of the SF-8 Health Survey. Lincoln (RI): Quality Metric Incorporated; 2001.
Items 5 to 8 (FIN): Schur S, Neubauer M, Amering M, Ebert-Vogel A, Masel EK, Sibitz I, Watzke H, Schrank B: Validation of the Family Inventory of Needs (FIN) for family caregivers in palliative care. Palliat Support Care 2015, 13(3):485-491.

Abbreviations: GAD-7, Generalized Anxiety Disorder Scale; PHQ-9, Patient Health Questionnaire – depression module; SF-8, short form of the Health Survey Form-36; FIN, Family Inventory of Needs
