# Supplementary material for: The CAREPAL-8: a short screening tool for multidimensional family caregiver burden in palliative care
Source: BMC Palliat Care. 2024 Aug 2;23:195. doi: 10.1186/s12904-024-01480-w (PMC11295689; doi:10.1186/s12904-024-01480-w)
Supplement: Supplementary file 3 — Supplementary Material 3 [file 12904_2024_1480_MOESM3_ESM.pdf]

Supplemental material

The CAREPAL-8: a short screening tool for multidimensional family caregiver burden in palliative care

Figure A3: Initial recommendations for family caregiver support

CAREPAL-8: Recommendations

Class 1: Currently stable family caregivers

Characterized by:
High distress, but: Low levels of psychopathological symptoms; sufficient resources (health-related quality of life, social support); sufficient need fulfillment.
Recommendation:
Support the family caregiver to the extent that he/she continues to do well. Holistic caregiver support by the multiprofessional palliative care team.

Class 2: Family caregivers with unmet needs

Characterized by:
High distress, but: Low levels of psychopathological symptoms; sufficient resources (health-related quality of life, social support); insuffient need fulfillment.
Recommendation:
Further assessment of the caregiver's palliative care needs. Evaluate and adress needs regarding basic information, information on treatment, support for the family caregiver himself/herself, patient comfort. Assessment can be supported by using the Family Inventory of Needs-questionnaire.

Class 3: Psychologically burdened family caregivers

Characterized by:
High distress, and: Increased risk of psychopathological symptoms; insufficient resources (health-related quality of life, social support); sufficient need fulfilment.
Recommendation:
Further asssessment of psychopathological symptoms (anxiety and depression). Assessment can be supported by the Generalized Anxiety Disorder Scale (GAD-7) and the Patient Health Questionnaire – Depression Module (PHQ-9). Assessment of resources regarding quality of life and availability of social support. To reduce the extent of pychological distress and improve resources, professional support by experts from the psychosocial field is required.

Class 4: High-risk family caregivers

Characterized by:
High distress, and: High risk of psychopathological symptoms; insufficient resources (health-related quality of life, social support); insufficient need fulfillment.
Recommendation:
Further assessment of psychopathological symptoms (anxiety and depression), resources regarding quality of life and availability of social support, and the palliative care needs of the family caregiver. To reduce the extent of pychological distress and improve resources, professional support by experts from the psychosocial field is required. Evaluate and adress needs regarding basic information, information on treatment, support for the family caregiver himself/herself, patient comfort.

Figure A3. Initial recommendations for family caregiver support according to the four family caregiver classes.
